# Cetuximab and Paclitaxel Drug Response in Head and Neck Tumor Stem Cells

**DOI:** 10.3390/biom15030352

**Published:** 2025-02-28

**Authors:** Vilson Serafim Júnior, Glaucia Maria de Mendonça Fernandes, Bianca Barbério Bogdan Tedeschi, Caroline Izak Cuzziol, Márcia Maria Urbanin Castanhole-Nunes, Letícia Antunes Muniz Ferreira, Gabriela Helena Rodrigues, Juliana Garcia de Oliveira-Cucolo, Érika Cristina Pavarino, Eny Maria Goloni-Bertollo

**Affiliations:** Genetics and Molecular Biology Research Unit [UPGEM], Faculty of Medicine of São José do Rio Preto [FAMERP], Avenida Brigadeiro Faria Lima, number 5416, Vila São Pedro, São José do Rio Preto 15090-000, SP, Brazil; vilson.serafim-junior@unesp.br (V.S.J.); fernandes_glaucia@hotmail.com (G.M.d.M.F.); biancabogdan04@hotmail.com (B.B.B.T.); carolizakcuzziol@outlook.com (C.I.C.); mcastanhole@gmail.com (M.M.U.C.-N.); leticiaamferreira@gmail.com (L.A.M.F.); rgabrielahelena@gmail.com (G.H.R.); juliana.cucolo@gmail.com (J.G.d.O.-C.); erika@famerp.br (É.C.P.)

**Keywords:** tumor stem cells, head and neck cancer, cancer treatment, chemotherapy, gene expression, cell signaling pathways

## Abstract

Head and neck cancer (HNC) is one of the most common types of cancer in the world, characterized by resistance to conventional therapies and an unfavorable prognosis due to the presence of tumor stem cells (TSCs). TSCs are cell subpopulations with high potential for invasion, migration, and metastasis, being responsible for the initiation and dissemination of cancer. This study aimed to evaluate the efficacy of treatments with cetuximab and paclitaxel, alone and in combination, in TSCs from oral cavity (SCC-28) and hypopharynx (FADU) cancer cell lines. In addition, the influence of the gene and protein expression of EGFR, *NTRK2* (TRKB), KRAS, and HIF-1α on the response to treatments was investigated. TSCs were identified based on ALDH staining, and cell viability assays (MTS) indicated that both TSCs and non-TSCs showed resistance to cetuximab monotherapy, while paclitaxel, either alone or in combination with cetuximab, was more effective in reducing cell viability. Real-time PCR and Western blot analysis revealed increased expression of KRAS and HIF-1α in TSCs, suggesting their possible association with treatment resistance. The results of this study point to specific molecular factors that influence therapeutic responses in HNC, with an emphasis on the efficacy of drug combinations to overcome TSC resistance. The identification of these molecular mechanisms may provide guidelines for the development of more targeted and effective therapies against HNC, improving clinical management and patient prognoses.

## 1. Introduction

Head and neck cancer (HNC) is one of the most common tumor types in the world [1]. According to data from the Global Cancer Observatory, in 2020, there were more than 1,518,000 new cases, with about 510,900 deaths, and it is estimated that by 2030 this number will reach 1,834,000 new cases and 643,400 deaths [2]. HNC is a heterogeneous group of tumors, including tumors of anatomical sites, such as the pharynx, larynx, sinuses, and oral cavity [3]. Treatment options for HNC include surgery, radiotherapy, and/or chemotherapy, and the choice of treatment varies with the tumor stage [4].

However, despite advances in drug therapy, HNC has a poor prognosis due to its low survival rate, metastasis, and tumor recurrence, which may be related to the presence of tumor stem cells (TSCs) [5,6]. TSCs are a small subset of cells with a high potential for invasion, migration, and metastasis and may be responsible for cancer initiation and dissemination [7].

The presence of TSCs is associated with a poor prognosis because it results in resistance to radiotherapy and chemotherapy and increases DNA repair, epithelial–mesenchymal transition, cell immortality, and tumor recurrence [8]. TSCs overexpress aldehyde dehydrogenase (ALDH), an enzyme group involved in the cell detoxification process and, therefore, can be used to identify TSC subsets present in the tumor [9,10,11].

In addition to the high expression of ALDH, other genetic and molecular factors may be related to the resistance of TSCs to drugs used in the treatment of HNC. The overexpression and activation of proteins involved to cell signaling pathways are directly related to the inefficiency of treatment against HNC. The epidermal growth factor receptor (EGFR), commonly overexpressed in epithelial tumors, is related to cell survival and resistance to apoptosis [12]. Another receptor that, when overexpressed, is related to tumor malignancy is Tropomyosin Related Kinase B (TRKB), encoded by the *NTRK2* gene [13].

When activated, EGFR and TRKB initiate a downstream cascade, activating other proteins, such as KRAS and HIF-1α, leading to self-renewal and resistance to treatment [14]. Therefore, the aim of this study was to evaluate the response of TSCs and non-TSCs to treatment with the drugs cetuximab and paclitaxel, used in the treatment of HNC, both as monotherapy and in combination. The present study also aimed to verify the differential expression of EGFR, TRKB, KRAS, and HIF-1α, involved in the tumor malignancy process in the subpopulations of TSCs and non-TSCs, and to verify whether the expression of these structures is related to the phenotype of TSCs, as well as the response of HNC to treatment.

## 2. Materials and Methods

### 2.1. Cell Culture

Cell culture was performed as shown in our previous work [10]. Briefly, larynx carcinoma (UM-SCC-28), called, from now on, only SCC-28 [15], and hypopharynx carcinoma (FADU) [16] cell lines were cultured in Dulbecco’s modified Eagle medium high glucose (DMEM; Sigma-Aldrich, St. Louis, MO, USA) supplemented with 10% fetal bovine serum (FBS; Gibco™, Carlsbad, CA, USA), Ham’s Nutrient Mixture F12 20% (Sigma-Aldrich), 1% L-glutamine (Gibco™), and 1% penicillin/streptomycin/amphotericin B (Gibco™) in a humidified atmosphere with 5% CO_2_ at 37 °C. After obtaining the required number of cells (approximately 1 × 10^6^), the cells were sent for separation into subpopulations of TSCs and non-TSCs.

### 2.2. Identification and Separation of TSCs via Cell Sorting

Using the specific intracellular biomarker ALDH alone (SCC-28) or ALDH associated with the CD133 biomarker (FADU), two subgroups of each cell line were identified as TSCs (positive for ALDH, or ALDH + CD133) and non-TSCs (no marker) through cell sorting on the BD FACSAria Fusion (BD Biosciences, Franklin Lakes, NJ, USA). Subsequent analyses were performed after cultivation, and the required numbers of TSCs and non-TSCs were obtained.

### 2.3. Confirmation of Stemness Properties

To confirm the stem cell separation process, the cell subgroups (TSCs and non-TSCs) of both SCC-28 and FADU cells were evaluated in triplicate using invasion, migration, and sphere-formation assays according to [11].

### 2.4. Invasion and Migration Assay

The quantitative analysis of invasive potential was performed using 24-well plates with BD Bio Coat Matrigel invasion chambers (BD Biosciences). Cells (2 × 10^4^) of each cell line were seeded in the upper compartment of the Transwell chamber, whereas the bottom chambers were filled with DMEM supplemented with 10% FBS. The plates were incubated for 24 h, and the cells that invaded or migrated into the lower membrane surface were fixed with 4% paraformaldehyde for 20 min and stained with crystal violet for 20 min. The cells were photographed under an inverted microscope and counted using ImageJ version 4.0 software. The migration assay followed the same protocol; however, 2 × 10^4^ cells from each cell line were seeded in Transwell chambers placed in 24-well plates without Matrigel.

### 2.5. Sphere-Forming Assay

TSCs have clonogenicity properties; thus, their capacity to generate tumor spheres was evaluated. Briefly, 2 × 10^4^ TSCs or non-TSCs of each cell line were passed through a mesh to ensure a single-cell suspension and seeded in low-adherence 6-well plates (ultra-low attachment plates, Corning Inc., Corning, NY, USA) with complete DMEM and incubated at 37 °C and 5% CO_2_ for 5 days. During this period, the culture medium was not changed. The cells were photographed at 0 h (immediately after plating) and after 120 h of cultivation. The colonies that formed were also counted using ImageJ version 4.0.

### 2.6. Treatment and MTS Assay

In total, 5 × 10^3^ cells of TSCs and non-TSCs of both cell lines were seeded in 96-well plates and treated with 0.06 mg/mL cetuximab, 0.05 mg/mL paclitaxel, or 0.06 mg/mL cetuximab combined with 0.05 mg/mL paclitaxel. After 24 h of treatment, the MTS assay was performed using the Cell Titer 96 Aqueous One Solution Cell Proliferation Assay (Promega, Madison, WI, USA) following the manufacturer’s instructions. Cell viability was determined by measuring the absorbance at 490 nm using an ELISA plate reader (Multiskan FC; Uniscience, São Paulo, Brazil). Untreated cells were used as controls, and the viability between treated and untreated cells was compared.

### 2.7. Gene Expression

The gene expression was evaluated to elucidate the mechanism involved in the drug response of both TSCs and non-TSCs. Actin β and GAPDH were used as endogenous controls. A non-TSC subpopulation was used as the calibrator (RQ = 1.0). The method used was 2-(Delta Ct) × 1000 Delta Ct = Ct of the target gene − (mean Ct of β-actin and GAPDH genes).

The RNA was extracted from 1 × 10^6^ cells using TRIzol reagent (Life Technologies, Carlsbad, CA, USA), according to the manufacturer’s instructions. The RNA concentration and quality were measured using a Qubit™ RNA HS Assay Kit with a Qubit^®^ 2.0 Fluorometer (Life Technologies). Reverse transcription was performed using a High-Capacity cDNA kit (Applied Biosystems, Foster City, CA, USA) according to the manufacturer’s instructions, and the final concentration of cDNA was 1 μg/μL. Quantitative PCR was performed using the TaqMan^®^ System (Life Technologies) and the StepOne Plus Real-Time PCR system 2.2.3 (Applied Biosystems), using TaqMan probes specific for *EGFR* (HS 01076090), *NTRK2* (HS 00178811), *KRAS* (HS 00364284), *HIF-1α* (HS 00153153), and two reference genes, *ACTB* (HS 01060665) and *GAPDH* (HS02758991). The reactions were performed in triplicate, using 1 μL of cDNA per well and included a negative control. Relative quantification (RQ) was performed using the 2^−ΔΔCt^ method, according to [17], after normalization to both reference genes; the non-TSC subset was used as the calibrator (RQ = 1.0).

### 2.8. Protein Expression

For protein expression (EGFR, TRKB, encoded by *NTRK2*, KRAS, HIF-1α, and β-actin) analyses, the total proteins were extracted using RIPA (Sigma-Aldrich), and their concentrations were estimated using the Pierce^TM^ BCA Protein Assay Kit (Thermo Fisher Scientific, Waltham, MA, USA), according to the manufacturer’s instructions. The expression levels of EGFR, TRKB protein (encoded by *NTRK2* gene), KRAS, HIF-1α, and β-actin were measured using Western blotting. Equal protein amounts (30 μg) were loaded onto 4–12% SDS-PAGE gels. After separation, the proteins were transferred using iBlotR Gel Transfer Stacks PVDF Regular (Invitrogen, Waltham, MA, USA). Blocking was performed for 1 h with 3% BSA in 0.5% Tris-buffered saline with 1% of Tween 20 (TBS-T). Primary antibodies, EGFR (Santa Cruz Biotechnology, Dallas, TX, USA), TRKB (Abcam, Cambridge, UK), KRAS (ANOVA), HIF-1α (Invitrogen), and β-actin (Sigma-Aldrich), at a dilution of 1:1000 in 3% BSA in 0.5% TBS-T, were then added and incubated overnight at 4 °C. Afterwards, the membranes were washed three times with TBS-T, and secondary anti-mouse (KRAS and β-actin) and anti-rabbit (EGFR, TRKB and HIF-1α) antibodies were used at a concentration of 1:20,000 in 3% BSA in 0.5% TBS-T and were incubated at room temperature for 1 h. Afterwards, the membranes were washed three times with TBS-T, and enhanced chemiluminescence reagent (Invitrogen) was used to detect immunoreactive secondary antibodies bound to the membrane. The images were captured using photo-documenter equipment, and the bands were measured using ImageJ version 4.0, as described by Hossein D 2017 [18].

### 2.9. Statistical Analysis

The results of the confirmation of the stemness properties were evaluated using *t*-tests. To verify the efficacy of drug treatments, cell viability data were evaluated using one-way RM ANOVA with a Bonferroni correction. RQ values for *EGFR*, *NTRK2*, *KRAS*, and *HIF-1α* were statistically analyzed. The continuous data distribution was evaluated using the D’Agostino-Pearson omnibus normality test. Statistical analyses were performed using GraphPad Prism version 9 (GraphPad Software, LLC, St. Diego, CA, USA); *p* ≤ 0.05 was considered statistically significant.

## 3. Results

### 3.1. Separation of SCC-28 and FADU Cells into TSC and Non-TSC Subsets

To obtain TSCs and non-TSCs subpopulations, cell sorting was initially performed. SCC-28 and FADU cells were sorted using the biomarker *ALDH* alone or associated with CD133. SCC-28 TSCs and non-TSCs represented 15.8% and 7.9% of the cells, respectively. FADU TSCs and non-TSCs represented 8.1% and 57.6% of the cells, respectively.

To ensure that the TSC and non-TSC groups were used, the other cells (63.3% of SCC-28 and 34.3% of FADU) were discarded in the intermediate region of the gates used in the FacsAria equipment, which could contain mixed TSCs and non-TSCs. Cell sorting data are available in Supplementary Material File S1.

### 3.2. ALDH-Positive Cells Have Greater Stemness Properties than Those of ALDH-Negative Cells

**Figure 1:**
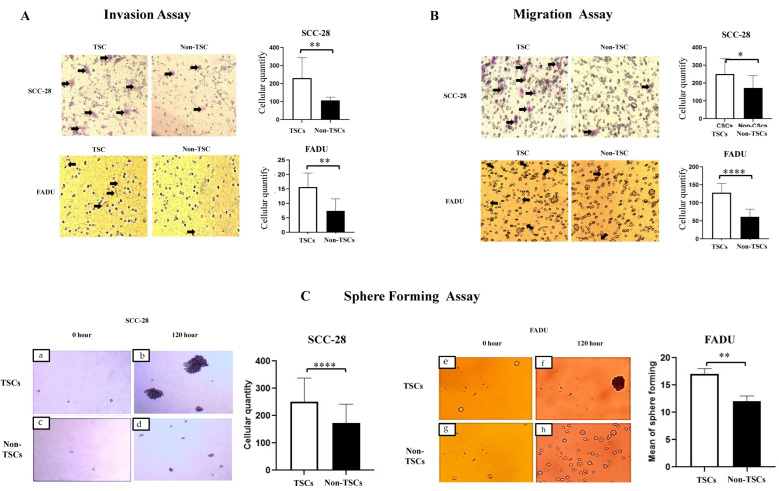
Confirmation of stemness properties in SCC-28 and FADU cells. The scales of pictures were captured using an optical microscope (×100), and the mean of cells in each group (TSCs and non-TSCs) was compared via a statistical analysis (**A**) Cell invasion assay. (**B**) Cell migration assay. (**C**) Tumor-sphere-formation assay. Cells were photographed at 0 h (SCC-28: (**a**,**c**); FADU: (**e**,**g**)) and after 120 h (SCC-28: (**b**,**d**); FADU: (**f**,**h**)) of cultivation. The mean of cells in each group (TSCs and non-TSCs) at 120 h was compared through a statistical analysis. Statistically significant differences were determined using the *t*-test. Note: * *p* < 0.050; ** *p* < 0.010; **** *p* < 0.0001.

Subsequently, to validate the separation process and confirm that the ALDH+ cells present a stem cell phenotype, invasion, migration, and sphere-formation assays were performed. The stemness property assay showed that SCC-28 and FADU TSCs had greater invasive potential (*p* < 0.0100 in both cell lines; Figure 1A). The same was observed for migration potential (SCC-28, *p* < 0.0500; FADU, *p* < 0.0001—Figure 1B). TSCs also showed significant clonogenicity with higher sphere-formation compared to those of non-TSCs (SCC-28, *p* < 0.0010; FADU, *p* < 0.0100—Figure 1C). These data confirm that the ALDH+ and ALDH− subpopulations have different behaviors and that ALDH+ presents a TSC phenotype.

### 3.3. The Combination of Cetuximab and Paclitaxel Is Efficient in Treating TSCs and Non-TSCs in HNC

**Figure 2:**
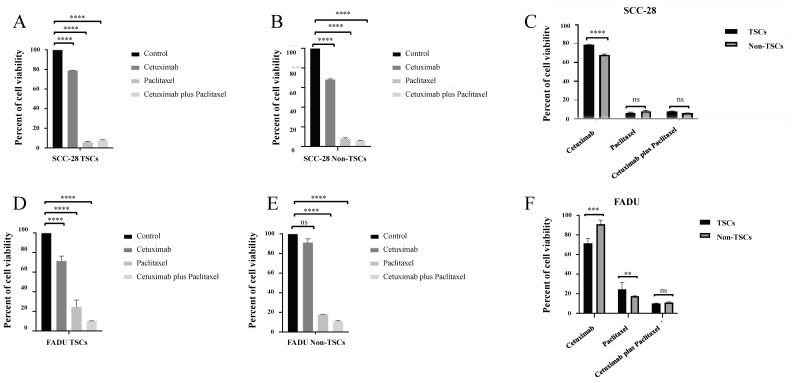
Cell viability after 24 h of treatment in both SCC-28 and FADU TSCs and non-TSCs after drug therapy. (**A**) Responses to treatment with cetuximab, paclitaxel, and cetuximab plus paclitaxel in the TSC subpopulation of the SCC-28 cell line compared with untreated cells (**B**) Responses to treatment with cetuximab, paclitaxel, and cetuximab plus paclitaxel in the non-TSC subpopulation of the SCC-28 cell line compared with untreated cells. (**C**) Comparison of response to treatment with cetuximab, paclitaxel, and cetuximab plus paclitaxel between TSCs and non-TSCs of the SCC-28 cell line. (**D**) Responses to treatment with cetuximab, paclitaxel, and cetuximab plus paclitaxel in the TSC subpopulation of the FADU cell line compared with untreated cells. (**E**) Responses to treatment with cetuximab, paclitaxel, and cetuximab plus paclitaxel in the non-TSC subpopulation of the FADU cell line compared with untreated cells. (**F**) Comparison of response to treatment with cetuximab, paclitaxel, and cetuximab plus paclitaxel between TSCs and non-TSCs of the FADU cell line. Note: ** *p* < 0.010; *** *p* < 0.001; **** *p* < 0.0001; ns: no significance.

After separation and confirmation of the phenotype of TSCs and non-TSCs, both subpopulations were treated with cetuximab, paclitaxel, and cetuximab plus paclitaxel to evaluate the response of the subpopulations to pharmacological treatment. In SCC-28 cells, cetuximab monotherapy was effective in eliminating TSCs and non-TSCs compared to the control group. TSCs and non-TSCs showed 79.1% (*p* < 0.0001) and 68.3% (*p* < 0.0001) viability, respectively, after 24 h of treatment compared with untreated cells. Alternatively, paclitaxel monotherapy was a more effective treatment for TSCs and non-TSCs in SCC-28 cells. After paclitaxel monotherapy, TSCs and non-TSCs showed 6.3% (*p* < 0.0001) and 7.9% (*p* < 0.0001) viability, respectively, compared with untreated cells. The combination of cetuximab and paclitaxel also effectively reduced SCC-28 cell proliferation after 24 h of treatment. Cell viability was 8% for TSCs (*p* < 0.0001) and 6% for non-TSCs (*p* < 0.0001). These data are presented in Figure 2A,B. Comparing the treatment effectiveness of cetuximab between both SCC-28 TSCs and non-TSCs, non-TSCs subpopulation showed a decrease in cell viability (*p* < 0.0001). On the other hand, after treatment using paclitaxel monotherapy or cetuximab plus paclitaxel, both SCC-28 TSCs and non-TSCs did not show statistical significance, as shown in Figure 2C.

In the FADU cell line, cetuximab monotherapy was effective in decreasing TSC viability to 71.4% (*p* < 0.0001) compared with untreated cells. On the other hand, it was ineffective for non-TSCs, which showed 91.3% viability (*p* > 0.0500) after 24 h of treatment compared to untreated cells. On the other hand, paclitaxel monotherapy was effective in reducing cell viability. After 24 h of treatment, the cell viability of TSCs was 24.2% (*p* < 0.0001) and that of non-TSCs was 17.9% (*p* < 0.0001) compared with untreated cells. In the FADU cell line, the combination of cetuximab and paclitaxel was the most effective treatment for reducing cell viability in both TSC and non-TSC groups. The cell viability of TSCs was 10.4% (*p* < 0.0001) and that of non-TSCs was 11.2% (*p* < 0.0001) compared with untreated cells. These data are presented in Figure 2D,E.

On the other hand, FADU non-TSCs showed enhanced viability compared with TSCs (*p* < 0.0010). Paclitaxel treatment was more effective in FADU non-TSCs than TSC populations (*p* < 0.0100). After treatment using cetuximab plus paclitaxel, both TSCs and non-TSCs of FADU cell lines did not show a difference, as shown in Figure 2F.

**Figure 3:**
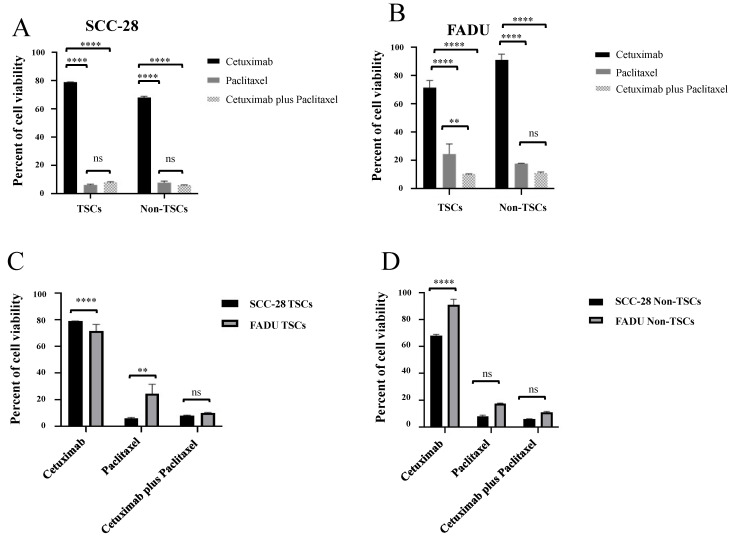
Comparison between treatments in each TSC and non-TSC population, as well as comparison of the therapeutic responses between both SCC-28 and FADU cell lines. (**A**) Comparison of the response of TSC and non-TSC subpopulations of the SCC-28 cell line to treatments with cetuximab, paclitaxel, and cetuximab plus paclitaxel. (**B**) Comparison of the response of TSC and non-TSC subpopulations of the FADU cell line to treatments with cetuximab, paclitaxel, and cetuximab plus paclitaxel. (**C**) Comparison of the response to treatment with cetuximab, paclitaxel, and cetuximab plus paclitaxel between TSC subpopulations of SCC-28 and FADU lines. (**D**) Comparison of response to treatment with cetuximab, paclitaxel, and cetuximab plus paclitaxel between non-TSC subpopulations of SCC-28 and FADU lines. Note: ** *p* < 0.010; **** *p* < 0.0001; ns: no significance.

Comparing the response between TSC groups, in SCC-28, paclitaxel monotherapy (*p* < 0.0001) and cetuximab plus paclitaxel (*p* < 0.0001) were more effective compared with cetuximab monotherapy. However, the difference between paclitaxel monotherapy and cetuximab plus paclitaxel was not statistically significant. In non-TSC subpopulations, the results were similar to data of TSCs. Cetuximab showed greater cell proliferation compared with paclitaxel monotherapy (*p* < 0.0001) and cetuximab plus paclitaxel (*p* < 0.0001), with data shown in Figure 3A.

In the FADU cell line, cetuximab monotherapy showed greater cell viability compared with paclitaxel monotherapy (*p* > 0.0001) and cetuximab plus paclitaxel (*p* < 0.0001) in both TSCs and non-TSCs. In addition, cetuximab plus paclitaxel was more effective than paclitaxel monotherapy in the TSC subpopulation (*p* < 0.0010), but in the non-TSC subpopulation, it did not show statistical significance. Data are shown in Figure 3B.

When comparing the treatment response between cell lines, SCC-28 TSCs showed more resistance to cetuximab monotherapy than FADU TSCs (*p* < 0.0001) but were more sensitive to paclitaxel monotherapy (*p* < 0.0100). Cetuximab plus paclitaxel treatment was not statistically significant, as shown in Figure 3C. On the other hand, SCC-28 non-TSCs were more sensitive to cetuximab treatment than FADU non-TSCs (*p* < 0.0001). Paclitaxel monotherapy and cetuximab plus paclitaxel treatment was not significant between both cell lines. Data are shown in Figure 3D.

### 3.4. EGFR, NTRK2, KRAS, and HIF-1α Exhibit Different Gene Expression Levels Between TSCs and Non-TSCs

The gene expression levels of *EGFR*, *NTRK2*, *KRAS,* and *HIF-1α* were evaluated to determine the differential expression profile between TSCs and non-TSCs and their association with the therapeutic response to cetuximab and paclitaxel.

**Figure 4:**
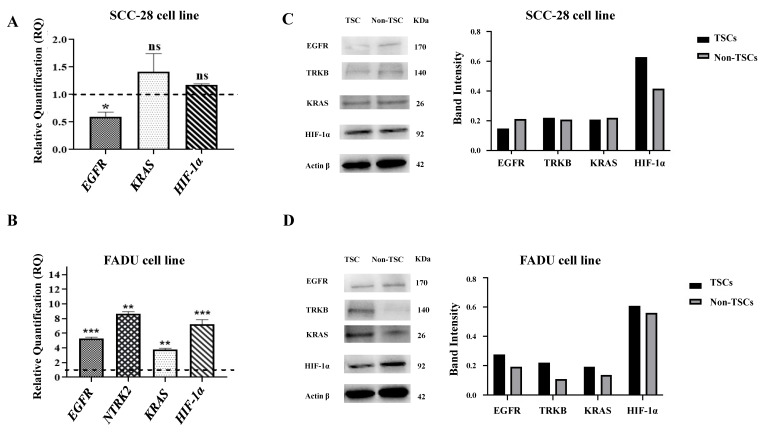
Relative gene expression levels and protein expression in SCC-28 and FADU cells. Data are presented as the RQ median and the comparison between TSCs and non-TSCs (median = 1). (**A**) SCC-28 gene expression of *EGFR*, *KRAS*, and *HIF-1α*. The *NTRK2* gene presented late expression in both TSCs and non-TSCs. (**B**) FADU gene expression of EGFR, *NTRK2*, *KRAS*, and *HIF-1α*. (**C**) SCC-28 Western blot analysis of EGFR, TrkB, KRAS, HIF-1α, and β-actin expression, and histogram showing the comparison between the protein band intensity for both TSCs and non-TSCs. (**D**) FADU Western blot analysis of EGFR, TrkB, KRAS, HIF-1α, and β-actin expression, and histogram showing the comparison between the protein band intensity for both SCC-28 TSCs and non-TSCs. The band intensity for TSCs and non-TSCs was measured using ImageJ and normalized to β-actin. Original figures can be found in Supplementary Materials File S2. Note: * *p* < 0.050; ** *p* < 0.010; *** *p* < 0.001; ns: no significance.

The SCC-28 TSCs showed downregulated *EGFR* compared with non-TSCs (RQ = 0.6254, *p* < 0.0500). On the other hand, *KRAS* and *HIF-1α* were slightly overexpressed (RQ = 1.4613 and RQ = 1.2240, respectively) in TSCs compared to non-TSCs; however, the difference was not statistically significant. The *NTRK2* gene showed late expression in both TSCs and non-TSCs; therefore, it was considered unexpressed. These data are presented in Figure 4A.

FADU TSCs showed overexpressed *EGFR* (RQ = 5.3378; *p* < 0.0001), *NTRK2* (RQ = 8.6806; *p* < 0.0010), *KRAS* (RQ = 3.7523; *p* < 0.0010), and *HIF-1α* (RQ = 7.2067; *p* < 0.0001) compared with those of non-TSCs. These data are presented in Figure 4B.

### 3.5. Protein Expression Levels of EGFR, TRKB, KRAS, and HIF-1α Are Different Depending on the Tumor Cell Line

In the SCC-28 cell line, Western blot analyses showed that EGFR protein levels were decreased in TSCs, and KRAS and HIF-1α were increased in TSCs according to gene expression. TRKB proteins levels did not differ between TSCs and non-TSCs. These data are presented in Figure 4C.

On the other hand, in the FADU cell line, EGFR, TRKB, KRAS, and HIF-1α showed increased protein levels in the TSC subset. These data are presented in Figure 4D.

## 4. Discussion

In head and neck squamous cell carcinoma (HNSCC), ALDH-positive cells are responsible for chemoresistance and radioresistance, increased mobility for metastasis, epithelial–mesenchymal transition, increased DNA repair, immortality, and tumor recurrence [8]. ALDH is a class of enzymes, with different isoforms, responsible for the metabolism of various xenobiotics. ALDH-positive cells can metabolize drugs rapidly, which can cause treatment inefficiency. These characteristics indicate that the presence of TSCs may be related to tumor aggressiveness, poor prognosis, metastasis, and resistance to drug therapy [7,8,19,20]. In turn, CD133 is a membrane glycoprotein, widely used in the characterization of TSCs. There is evidence in the literature that demonstrates that CD133 is associated with the tumorigenic potential of TSCs, in addition to conferring greater survival and resistance to antitumor treatment in this subpopulation [21]. In the present study, it was possible to observe that both cells sorted using ALDH alone and cells sorted using ALDH + CD133 presented the characteristics of TSCS. In our previous study [11], cells sorted using different combinations of markers and also ALDH alone showed similar stemness potential, corroborating data with the current study.

**Figure 5:**
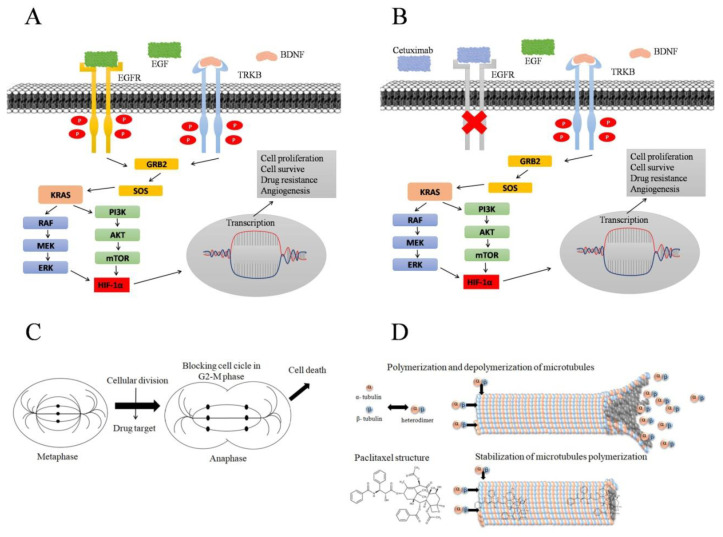
Signaling pathway activated by EGFR and TRKB receptors resulting in cell proliferation. (**A**) TK receptors are activated by endogenous binding, initiating a cascade downstream of cell proliferation. After binding to the growth factor, TK receptors are phosphorylated and activate KRAS, which can activate HIF-1A via the MAPK or PI3K pathways. HIF-1A induces gene transcription leading to cell proliferation, cell survival, drug resistance, and angiogenesis. Adapted by Amatu et al., 2016 [25,43]. (**B**) EGFR and TRKB crosstalk; cetuximab drug binding and EGFR receptor inhibition, blocking EGFR-dependent downstream cascade. TRKB activation, initiating cell proliferation processes, even when cetuximab treatment inhibits EGFR. Adapted by Gotz and Sentner, 2014 [23]. (**C**) Process of cell proliferation and targeting of drugs that act on cellular microtubules, blocking the cellular division process in the G2–M phase in the anaphase stage, causing cell death. Adapted by Souza, MVN 2004 [41]. (**D**) Polymerization and depolymerization process of alpha and beta tubulin heterodimers, forming cell microtubules, which are crucial for cell division; paclitaxel binding in alpha–beta tubulin heterodimers, leading to microtubule stabilization and causing cell death. Adapted by Souza, MVN 2004 [41] and Sueth-Santiago, V et al., 2017 [44].

In addition to drug resistance, we believe that the greater tumorigenic potential of TSCs is due to increased expression of genes and proteins related to cell proliferation. EGFR and TRBK are tyrosine kinases that, after binding to growth factors, lead to KRAS activation [22,23]. KRAS, in turn, leads to the activation of HIF-1a through the mitogen pathways activated by protein kinase or phosphatidylinositol 3 kinase, leading to cell division, metastatic behavior, drug resistance, and tumor recurrence, associated with poor prognosis [24,25] (Figure 5A). Activation of the cell signaling pathway gives the cells characteristics similar to those presented in TSCs, which may indicate a relationship between the expression of these genes and proteins and the stem cell phenotype.

SCC-28 TSCs showed decreased EGFR expression, and after treatment with cetuximab monotherapy, cell viability was enhanced compared with that in non-TSCs. Alternatively, FADU TSCs showed EGFR overexpression, and after treatment, decreased cell viability was observed compared with that in non-TSCs. These data indicate that the response to cetuximab treatment depends not only on the presence or absence of TSCs but also on the status of EGFR expression in tumor cells [26,27].

Cetuximab is a monoclonal antibody of the IgG1 isotype that has a greater affinity for EGFR than its endogenous ligand epidermal growth factor [28] (Figure 5B). The majority of squamous cell carcinomas arising in the upper airway show EGFR overexpression; therefore, cetuximab is indicated for the treatment of this tumor type [29,30]. Tumors with TSCs are resistant to cetuximab; however, the mechanisms underlying this resistance remain unclear [31,32]. A possible explanation for the resistance of cells to cetuximab is the presence of other tyrosine kinase receptors, which can activate the same signaling pathway. Even with EGFR inhibition, activation of the downstream pathway leads to cell proliferation.

Moreover, in our study, the SCC-28 TSCs, FADU TSCs, and SCC-28 non-TSCs were sensitive to cetuximab monotherapy compared to those in untreated cells. FADU non-TSCs were resistant to cetuximab. Similar EGFR expression levels were observed between SCC-28 TSCs and FADU non-TSCs, but subpopulations showed different drug responses to cetuximab monotherapy. These data suggest that EGFR expression leads to cell sensitivity levels but is not the only factor involved in the cetuximab monotherapy response in TSCs and non-TSCs, as reported by Silva Galbiatti-Dias (2018) [33], who noted a difference in drug responses between different anatomic sites.

Furthermore, the expression of other genes and proteins related to cell division may contribute to cetuximab treatment ineffectiveness. TRKB can be transactivated through EGFR via a crosstalk mechanism; even with the inhibition of this receptor, the cell proliferation cascade is activated as described by Gotz et al., 2014 [23] (Figure 5B). A study evaluating the effects of certain drugs in a colorectal cancer model demonstrated that the combination of K252a (a TRKB inhibitor drug) and cetuximab was more efficient than cetuximab monotherapy [14]. In the SCC-28 cell line, TRKB is not expressed and both TSCs and non-TSCS were sensitive to cetuximab monotherapy treatment, highlighting the relationship between the profile of TRKB expression and the response to cetuximab monotherapy. These data corroborate the findings described in our previous work [10] where it was also noted that TRKB expression was undetected in the laryngeal cancer cell line HEp-2, which suggests that TRKB is not commonly expressed in all HNC anatomic sites.

KRAS expression is another factor that can lead to resistance to drug responses. De Roock et al. (2008) demonstrated that *KRAS* overexpression induces resistance to cetuximab treatment in colorectal cancer [34]. In HNSCC, the overexpression of KRAS and other RAS family members is also associated with cetuximab resistance [35]. The KRAS mutation is another factor related to drug resistance, because when constitutively activated, KRAS can stimulate cell proliferation without growth factor activation, even at low expression levels [36], and the reduction in KRAS expression through the use of miRNAs decreases the tumorigenic potential of cancer cells [37], demonstrating that KRAS plays a pivotal role in cancer cell survival and proliferation. In the present study, it was possible to observe that KRAS expression was increased in TSCs of both cell lines, but the response to cetuximab, in monotherapy, was different in each of them, indicating that the KRAS expression profile does not directly influence the response to cetuximab monotherapy but that it is directly related to the expression of HIF-1α.

Studies on the cystic adenoid salivary gland and gastric cancer have shown that HIF-1α expression and hypoxic conditions are common in these tumor models and that this characteristic is related to the level of aggressiveness in these tumors, in addition to their resistance to treatment [38,39]. On the other hand, Wiechec et al., 2017 [7] showed that HIF-1α overexpression leads to an improvement in the response to cetuximab treatment in the oral cavity, tongue, and larynx cancer cell lines. In the present study, increased HIF-1α overexpression may be responsible for sensitizing FADU TSCs to cetuximab, corroborating the data. HIF-1α, as a transcription factor, when activated, can increase EGFR expression, which consequently sensitizes cells to cetuximab. In addition, HIF-1a stimulates angiogenesis, producing abnormal blood vessels, favoring the targeted delivery of drugs to the tumor environment [40]. The increase in EGFR expression caused by HIF1a expression, associated with targeted drug delivery to the tumor, may justify the sensitization to cetuximab.

In the present study, we found that TSCs and non-TSCs of both SCC-28 and FADU demonstrated sensitivity to paclitaxel treatment compared to untreated cells. Paclitaxel inhibits cell proliferation in the G2–M phase in the anaphase stage [41] (Figure 5C). Paclitaxel binds to cellular microtubules, leading to the inhibition of mitotic spindle formation by stabilizing these microtubules, preventing the polymerization and depolymerization of alpha-beta tubulin dimers, blocking cell proliferation, and causing cell death [42] (Figure 5D). Therefore, in our study, paclitaxel monotherapy appeared to be more effective than cetuximab. Even if the downstream signaling cascade is not blocked by cetuximab or KRAS constitutive activation, cell proliferation is interrupted, thereby decreasing cell viability.

A study conducted on uterine endometrial cancer showed that cells with high ALDH expression were resistant to paclitaxel treatment and that after the inhibition of ALDH with specific drugs, these cells were sensitized to treatment [45]. In the present study, there was no statistically significant difference between the viability of TSCs and non-TSCs in the SCC-28 cell line after paclitaxel monotherapy. On the other hand, FADU TSCs treated with paclitaxel monotherapy showed greater cell viability compared with FADU non-TSCs indicating that paclitaxel was more effective in eliminating non-TSCs at this tumor site, thus corroborating the study mentioned above.

In triple-negative breast cancer, TSCs with high TRKB expression are responsible for tumor recurrence after paclitaxel treatment [46]. In the present study, paclitaxel monotherapy decreased the viability of both TSCs and non-TSCs with TRKB expression but did not eliminate all tumor cells, which can cause tumor recurrence. Another reason for the greater viability after paclitaxel monotherapy treatment may be KRAS overexpression in FADU TSCs. Amatu et al. (2016) reported that KRAS activation leads to the activation of another cell compound that stimulates cell proliferation [25] corroborating data from our study. The drug response to paclitaxel can also be related to the RAS profile.

Galbiatti-Dias et al., 2018, found that oral cavity TSCs present greater cell viability after paclitaxel treatment; however, laryngeal TSCs showed decreased cell viability compared with non-TSCs but without statistical significance [33]. In the present study, there was no statistical significance between SCC-28 TSCs and non-TSCs, but TSCs presented a greater decrease in cell viability than non-TSCs after paclitaxel monotherapy.

Comparing paclitaxel monotherapy with cetuximab plus paclitaxel, we did not find statistical significance both in TSCs and non-TSCs of SCC-28 and FADU cell lines. These data suggest that paclitaxel monotherapy is efficient in reducing the viability of TSCs and non-TSCs and that its association with cetuximab, in the present study, does not have a great therapeutic contribution. On the other hand, the effects of combined cetuximab and paclitaxel treatment were similar to those of platinum and cetuximab combination therapy [47]. The combination of cetuximab and paclitaxel is well accepted and tolerated and has demonstrated effects in patients with recurrent/metastatic HNC with platinum intolerance [11,48]. Moreover, the combination of cetuximab and paclitaxel has better effects than those of the combination of methotrexate and celocoxinib, improving the overall survival of patients with HNSCC [49]. One explanation for the different findings is the fact that, in the present study, cell lines from non-metastatic tumors were used, in addition to the fact that primary tumors are more representative than cell lines.

When compared with cetuximab monotherapy and paclitaxel monotherapy, cetuximab plus paclitaxel was more effective in decreasing the proliferation of SCC-28 non-TSCs and both FADU TSCs and non-TSCs. According to Harada et al. (2014), this is because the combination of these drugs inhibits NF-κB in HNSCC [50].

The greater cell viability after cetuximab plus paclitaxel treatment in SCC-28 TSCs may be due to the EGFR status in this subset [27]. Although there was no statistical difference between the groups, these data reinforce that there are differences between the responses to the same therapeutic strategy, depending on the tumor site and molecular profile.

## 5. Conclusions

Finally, we concluded that the use of paclitaxel both in monotherapy and associated with cetuximab significantly reduced the cell viability of HNC cell lines. Although the increased expression of ALDH may influence the response to treatment, other points must be taken into account, such as the molecular profile, both in the TSC population and in the non-TSC population, since the expression of EGFR, TRKB, KRAS, and HIF-1α, although not shown to be related to the tumorigenic abilities of TSCs, has been shown to influence the response to treatment. However, for a better understanding of these findings, it is important to carry out studies on primary tumors, as these would be more representative of the physiological and molecular aspects of the general population.

## Data Availability

For the present study, all data are available in the manuscript, in the form of results. The images that were cut for the manuscript have their original versions available in the supplementary material.

## Supplementary Materials

The following supporting information can be downloaded at: https://www.mdpi.com/article/10.3390/biom15030352/s1, File S1: Separation* of SCC-28 and FADU cells into TSC and non-TSC subsets*. File S2: Western Blotting images.
