# Supplementary material for: Cetuximab and Paclitaxel Drug Response in Head and Neck Tumor Stem Cells
Source: Biomolecules. 2025 Feb 28;15(3):352. doi: 10.3390/biom15030352 (PMC11940455; doi:10.3390/biom15030352)
Supplement: Supplementary file 1 [file biomolecules-15-00352-s001.zip › Suplementary material - File S1.pdf]

SUPLEMENTARY MATERIAL

File S1: Separation *of SCC-28 and FADU cells into TSC and non-TSC subsets*

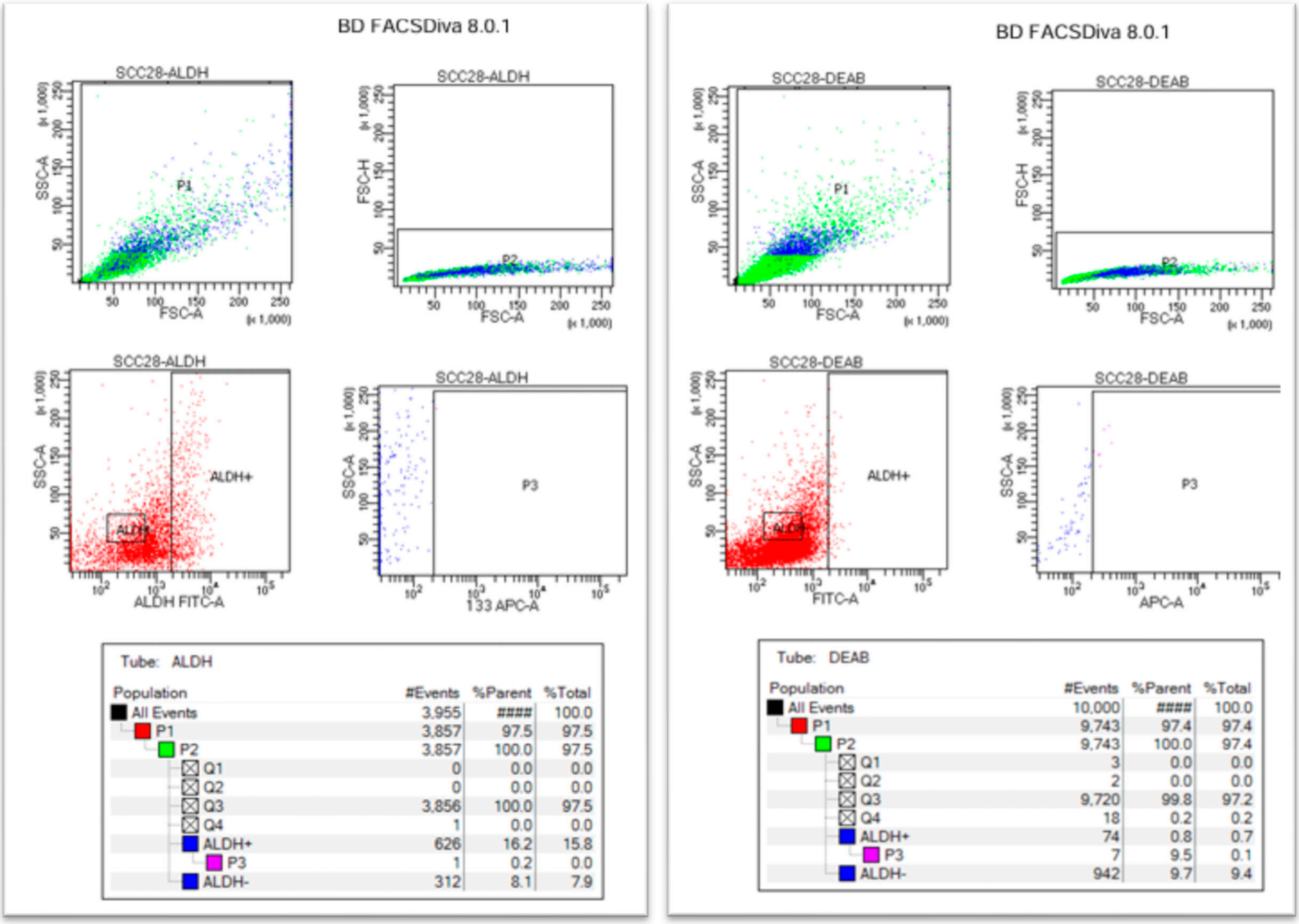

Tube: ALDH

Population	#Events	%Parent	%Total
All Events	3,955	####	100.0
P1	3,857	97.5	97.5
P2	3,857	100.0	97.5
Q1	0	0.0	0.0
Q2	0	0.0	0.0
Q3	3,856	100.0	97.5
Q4	1	0.0	0.0
ALDH+	626	16.2	15.8
P3	1	0.2	0.0
ALDH-	312	8.1	7.9

Tube: DEAB

Population	#Events	%Parent	%Total
All Events	10,000	####	100.0
P1	9,743	97.4	97.4
P2	9,743	100.0	97.4
Q1	3	0.0	0.0
Q2	2	0.0	0.0
Q3	9,720	99.8	97.2
Q4	18	0.2	0.2
ALDH+	74	0.8	0.7
P3	7	9.5	0.1
ALDH-	942	9.7	9.4

FACSDiva Version 6.1.1

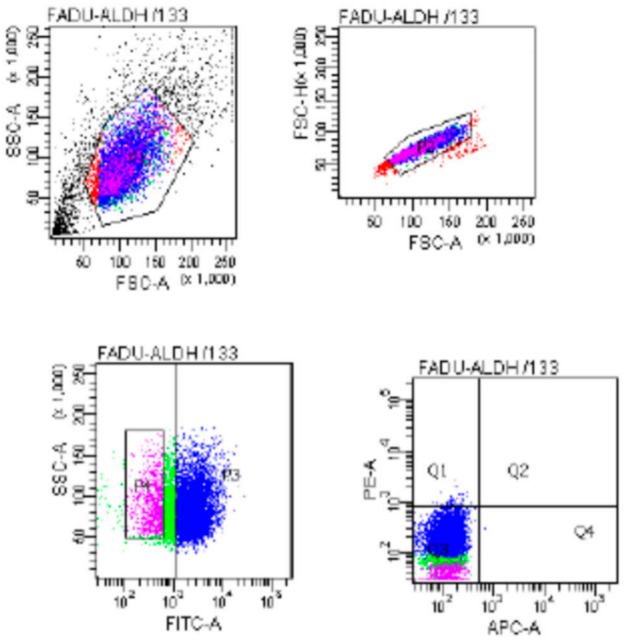

Tube: ALDH /133

Population	#Events	%Parent	%Total
All Events	10,000	####	100.0
P1	8,210	82.1	82.1
P2	7,745	94.3	77.4
P3	5,758	74.3	57.6
Q1	50	0.6	0.5
Q2	0	0.0	0.0
Q3	7,694	99.3	76.9
Q4	1	0.0	0.0
P4	808	10.4	8.1

FACSDiva Version 6.1.1

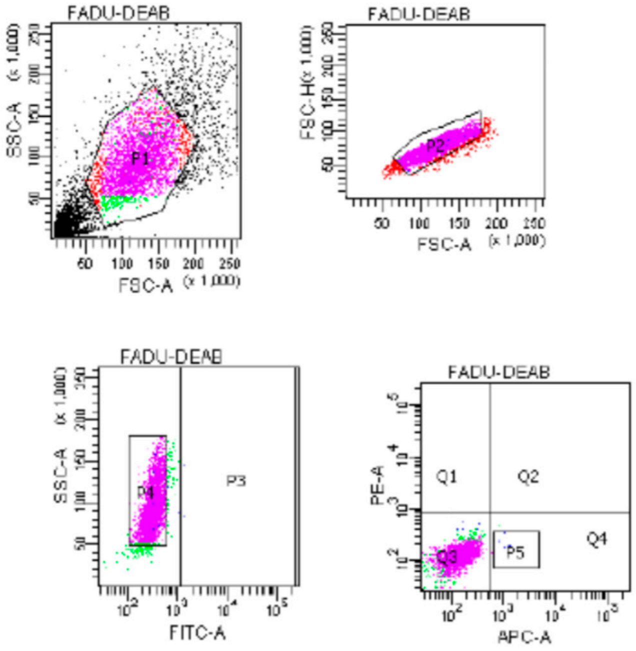

Tube: DEAB

Population	#Events	%Parent	%Total
All Events	10,000	####	100.0
P1	3,679	36.8	36.8
P2	3,175	86.3	31.8
P3	5	0.2	0.0
Q1	0	0.0	0.0
Q2	0	0.0	0.0
Q3	3,164	99.7	31.6
Q4	11	0.3	0.1
P4	2,948	92.9	29.5
P5	8	0.3	0.1
