# Supplementary material for: Cetuximab and Paclitaxel Drug Response in Head and Neck Tumor Stem Cells
Source: Biomolecules. 2025 Feb 28;15(3):352. doi: 10.3390/biom15030352 (PMC11940455; doi:10.3390/biom15030352)
Supplement: Supplementary file 1 [file biomolecules-15-00352-s001.zip › Suplementary material - File S2.pdf]

SUPLEMENTARY MATERIAL

File S2: Western Blotting images

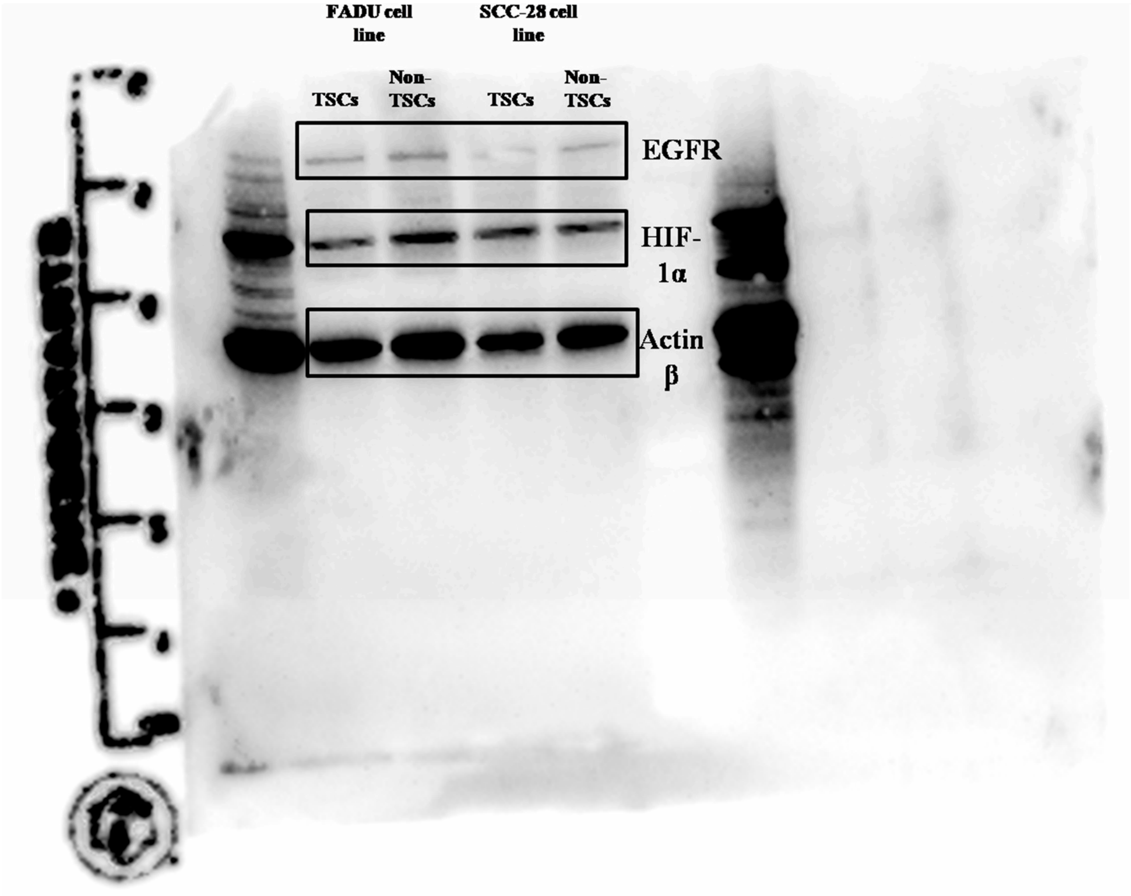

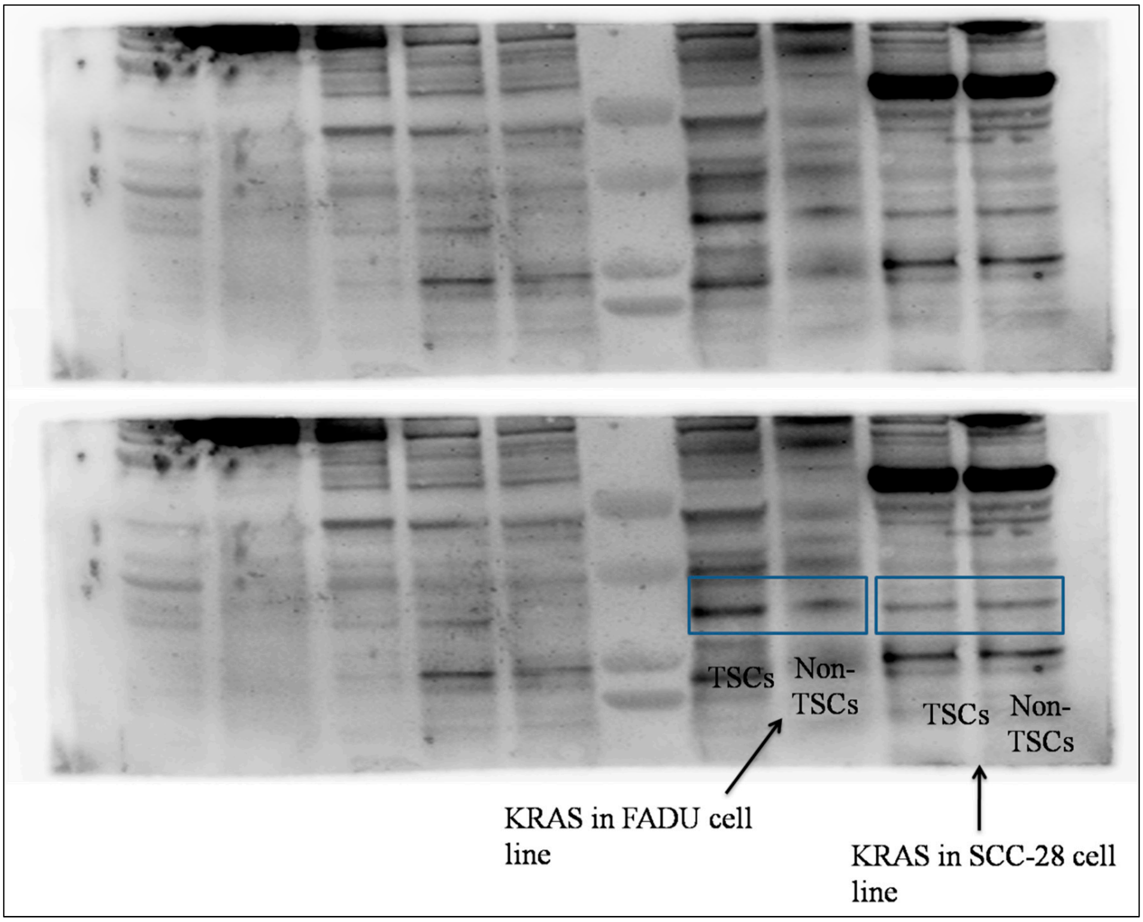
TSCs
Non-TSCs
TSCs
Non-TSCs
KRAS in FADU cell line
KRAS in SCC-28 cell line

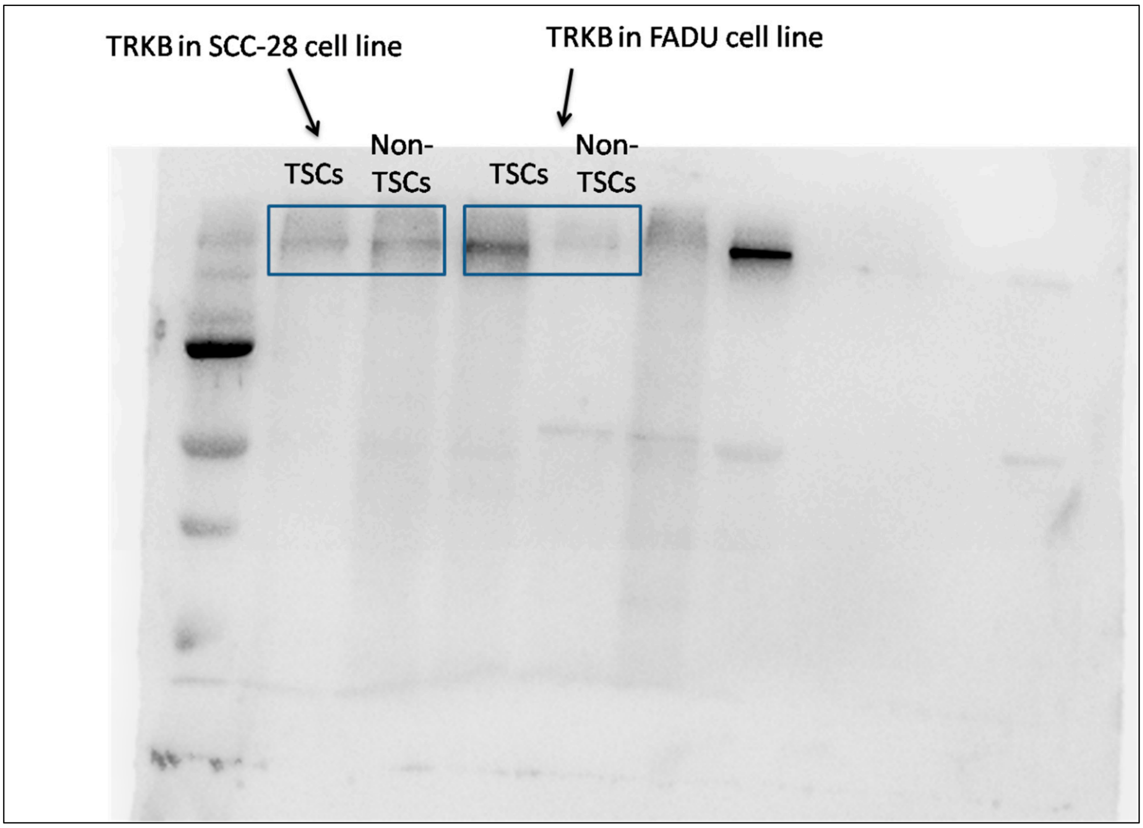
TRKB in SCC-28 cell line
TRKB in FADU cell line
TSCs
Non-TSCs
TSCs
Non-TSCs
